# Coriander leaf essential oil as an immunomodulator: Enhancing *NF-κB*-driven RAW246.7 murine macrophages response to *Candida albicans*

**DOI:** 10.1371/journal.pone.0337300

**Published:** 2026-04-17

**Authors:** Pratsanee Hiengrach, Pasinee Sangsiwarit, Waewta Kuwatjanakul, Kittipan Samerpitak, Pitchaya Luksanawilas

**Affiliations:** 1 Department of Microbiology, Faculty of Medicine, Khon Kaen University, Khon Kaen, Thailand; 2 Research and Diagnostic Center for Emerging Infectious Diseases (RCEID), Faculty of Medicine, Khon Kaen University, Khon Kaen, Thailand; 3 Clinical Microbiology Unit, Clinical Laboratory Section, Srinagarind Hospital Faculty of Medicine, Khon Kaen University, Khon Kaen, Thailand; 4 Department of Microbiology, Faculty of Medicine, Chulalongkorn University, Bangkok, Thailand; Cleveland Clinic Lerner Research Institute, UNITED STATES OF AMERICA

## Abstract

Coriander is a cultivated aromatic herb used as a culinary ingredient and in traditional medicinal treatments worldwide. Coriander essential oil contains various bioactive constituents with antibacterial, antioxidant, and anti-inflammatory effects. The oil also has antifungal activity against *Candida albicans,* the most common opportunistic fungal pathogen causing infections at various body sites. Furthermore, the oil can increase macrophage phagocytic activity. However, its effect on other macrophage functions during *C. albicans* infection is still unclear. This study aims to investigate the effect of coriander leaf essential oil on macrophage activity via the *NF-κB* gene expression during *C. albicans* infection. RAW264.7, a murine macrophage cell line, was cultured with viable *C. albicans*, either in the absence or presence of the oil (0 − 50 μg/mL). The fungal killing activity, pro- and anti-inflammatory cytokine production, and *NF-κB* gene expression were assessed. Our results revealed the potential of coriander leaf essential oil as an immunomodulator that enhances macrophage responses to *C. albicans* via the activation of the *NF-κB* gene. These findings may help to further the development of coriander leaf essential oil as an adjuvant antifungal and immunomodulatory medication.

## Introduction

Coriander (*Coriandrum sativum*), also known as Chinese parsley, is an aromatic and therapeutic annual plant belonging to the Apiaceae family [1]. It is widely cultivated across a diverse range of climatic regions worldwide. In Thai cuisine, fresh coriander leaves are used as a garnish for rice dishes, soups, and curries. Coriander has been reported to have potential in the traditional medicinal therapies of several health conditions, such as anxiety, constipation, diabetes mellitus, dyspepsia, parasitic infection, irritable bowel syndrome (IBS), and dermatological irritation [2,3]. Coriander essential oil, typically extracted from the dried seeds or leaves via steam distillation, has been reported to have antimicrobial properties that prevent bacterial contamination in food. Additionally, it contains bioactive compounds with antioxidant and anti-inflammatory effects [4]. Previous studies have demonstrated that coriander essential oil exerts potent antifungal activity against *Candida* species, particularly *C. albicans* [5].

Approximately 50% of the population has *C. albicans* as a common commensal fungus that colonizes the skin, gastrointestinal tract, vaginal tract, and oral cavity. As an opportunistic pathogen that proliferates when host-microbe balance is disrupted by immunological dysfunction, microbiota dysbiosis, or barrier degradation, *Candida* is an increasing global health concern, particularly in immunocompromised and critically ill patients [6,7]. Clinical manifestations range from superficial mucocutaneous infections to life-threatening invasive disease [8]. Virulence traits include yeast-to-hyphae transition, adhesion to host tissues and medical devices (adhesins, ALS family), secretion of hydrolytic enzymes (aspartyl proteinases, phospholipases, and lipases), evasion of host immune responses, and antifungal tolerance/resistance [8–10]. Recently, *Candida* infections frequently resulted in therapeutic failure, especially with azole drugs, largely due to antifungal resistance mediated by multiple mechanisms, such as biofilm formation [11,12].

Interestingly, the coriander essential oil binds to ergosterol in the fungal cell membrane, leading to increased ionic permeability, membrane disruption, and cell death. Moreover, it has been shown to inhibit *Candida* biofilm formation, thereby reducing fungal adherence and pathogenicity [13]. Additionally, coriander essential oil has been reported ability to modulate host immune responses, enhancing the phagocytic capacity of macrophages, the key innate immune cells responsible for the early recognition and elimination of fungal pathogens. However, the influence of the oil on other macrophage functions, such as fungal killing activity and cytokine-mediated inflammatory mechanisms during *C. albicans* infection remains unclear. Therefore, this study aims to investigate the effects of coriander essential oil on macrophage function and *NF-κB* gene expression during *C. albicans* infection. The results may offer novel insights into the potential use of coriander essential oil as a natural adjuvant for fungal infection therapy.

## Methods

### Ethical approvement

The research protocol employed in this study was reviewed and approved by the Center for Ethics in Human Research, Khon Kaen University (ECKKU), Thailand. Ethical approval was granted under the reference number HE671565.

### Cultivation of murine macrophage RAW264.7 cells

The RAW264.7 murine macrophage cell line was maintained in RPMI 1640 medium (Thermo Fisher Scientific, Waltham, MA, USA), supplemented with 10% (v/v) heat-inactivated fetal bovine serum (FBS) (Sigma-Aldrich, St. Louis, MO, USA) and 2 mM L-glutamine. The cells were incubated at 37°C in a humidified atmosphere containing 5% CO₂ [14].

### Cultivation of *C. albicans*

*C. albicans* strain ATCC90028 (Fisher Scientific, Waltham, MA, USA), obtained from the American Type Culture Collection, was cultured on Sabouraud dextrose agar (SDA; Oxoid, Basingstoke, Hampshire, UK) at 37°C for 48 h [15]. Then, the yeast cells were suspended in 1x phosphate-buffered saline (PBS) at pH 7.4, and the final concentration was adjusted to 1 × 10⁵ CFU/mL before experimentation.

### Liquid chromatography-mass spectrometry of coriander leaf essential oil

Laboratory-grade coriander leaf essential oil was obtained from Nature in Bottle Co., Ltd. (New Delhi, India). The separation and quantification of its constituents were performed using a liquid chromatography-mass spectrometry (LC-MS) system (U2Bio, Thailand) using a modified published protocol [16]. In brief, 150 µL of coriander leaf essential oil was mixed with 150 µL of 70% methanol (MeOH) containing 100 ng/mL sulfadimethoxine as an internal standard. The mixture was centrifuged at 14,000 rpm for 10 min, and the supernatant was transferred to an LC-MS vial for analysis. Chromatographic separation was carried out using a Poroshell 120 EC-C18 column (2.1 × 100 mm, 2.7 µm) maintained at 50°C. The injection volume was 10 µL. The mobile phases consisted of 0.1% formic acid in water (solvent A) and 0.1% formic acid in acetonitrile (solvent B), with a flow rate of 0.4 mL/min.

### Evaluation of macrophage-associated parameters

#### Measurement of fungicidal activity.

To evaluate the candidacidal activity of the macrophages, RAW264.7 cells were seeded into a 96-well microplate at a density of 1 × 10⁴ cells/well. Following overnight incubation, the viable *C. albicans* was co-incubated with the macrophages at 37°C in a 5% CO₂ atmosphere for 0, 6, and 18 h, either in the absence or presence of coriander essential oil (0 − 50 μg/mL). After each incubation period, 100 µL of the co-culture was plated onto SDA and incubated at 37°C for 72 h. The *Candida* colonies were subsequently enumerated to assess macrophage-mediated killing activity [17].

### Measurement of pro- and anti-inflammatory cytokines

To investigate the cytokines secreted by the macrophages, RAW 264.7 cells were seeded into a 96-well microplate overnight at a density of 1 × 10⁴ cells*/*well. Then, live *Candida* yeast cells were co-incubated at 37ºC under 5% CO_2_, incubated for 0, 6, and 18 h, with or without coriander extract oil (0 − 50 μg/mL). Next, the supernatant was collected to determine of the amount of pro-inflammatory cytokines (IL-6 and TNF-α) and anti-inflammatory cytokines (IL-10) using enzyme-linked immunosorbent assay (ELISA; Invitrogen, Massachusetts, USA). All the cytokines were quantified by spectrophotometry at excitation and emission wavelengths of 450 and 570 nm, respectively [18,19].

### Measurement of gene-associated inflammatory pathway

To investigate the inflammatory signaling pathway in the RAW264.7 macrophages, the expression of *NF-κB* was evaluated at the transcriptional level using quantitative reverse transcription polymerase chain reaction (qRT-PCR). RAW264.7 cells were seeded into 96-well microplates at a density of 1 × 10⁴ cells per well and incubated overnight. Subsequently, viable *C. albicans* cells were co-incubated with the macrophages at 37°C under humidified 5% CO₂, incubated for 0, 6, and 18 h, either in the absence or presence of coriander leaf essential oil (0 − 50 μg/mL). Macrophages were collected from each condition for RNA extraction using TRIzol reagent (Invitrogen, Massachusetts, USA), following the manufacturer’s protocol [20]. Complementary DNA (cDNA) was synthesized using a high-capacity cDNA reverse transcription kit (Thermo Fisher Scientific, Massachusetts, USA). The qRT-PCR was performed using Maxima SYBR Green qPCR Master Mix (Thermo Fisher Scientific) on a CFX96 touch real-time PCR detection system (Bio-Rad, California, US). Gene expression levels were analyzed using the ΔCt method, following a published method [21]. The following primers were used for *NF-κB*: forward, 5’-AGCCAGCTTCCGTGTTTGTT-3’ and reverse, 5’-AGGGTTTCGGTTCACTAGTTTCC-3’ [21]. Experiments were repeated three times.

### Statistical analysis

Data are presented as mean ± standard error (SE). Differences between groups were examined for statistical significance by one-way analysis of variance (ANOVA) followed by Tukey’s analysis. All statistical analyses were performed with GraphPad Prism version 9.0 software (Boston, USA). A *p-value* of ≤ 0.05 was considered statistically significant.

## Results

### Constituents of coriander leaf essential oil

**Fig 1:**
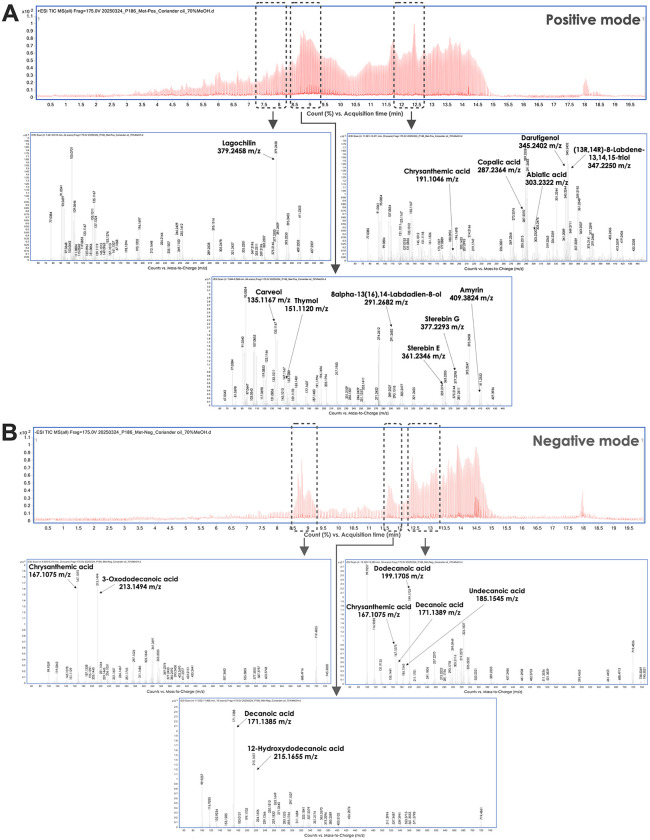
A chromatography of coriander leaf essential oil analyzed by LC-MS, showing results in positive ion mode (upper panel) and negative ion mode (lower panel).

Coriander leaf essential oil has been identified as a rich source of diverse phytochemical compounds, including both identified and unidentified compounds. The identified compounds included various mono-, di-, and triterpenoids, fatty acids, and naphthofuran derivatives (Table 1). Among the monoterpenoids detected were thymol, carveol, and chrysanthemic acid. The essential oil also contained several di- and triterpenoids, such as darutigenol, lagochilin, amyrin, darutigenol, sterebin E, sterebin G, and abietic acid, along with a range of labdane- and abietane-type diterpenoids. In addition to terpenoids, coriander leaf essential oil was found to contain a complex mixture of fatty acids. Saturated fatty acids, such as lauric acid, capric acid, decanoic acid, undecanoic acid, dodecanoic acid, and hydroxydodecanoic acid, were identified. Moreover, several bioactive oxylipins, including 3-oxo-dodecanoic acid, 11-hydroxyeicosatetraenoic acid (11-HETE), 9-hydroxyeicosapentaenoic acid (9-HEPE), and 12-oxo-eicosatetraenoic acid (12-OxoETE), were detected (Fig 1A and Fig 1B).

**Table 1:** Phytochemical profile of coriander leaf essential oil using the LC-MS method.

Mode	Average *m/z*	Metabolite name	Ontology	Total score	S/N average
pos	409.38245	Amyrin	Triterpenoids	1.06	1,993.61
pos	379.24524	Lagochilin	Diterpenoids	1.22	1,442.98
pos	377.22992	Sterebin G	Colensane and clerodane diterpenoids	0.90	53.72
neg	365.26691	Nervonic acid	Very long-chain fatty acids	1.01	184.67
pos	361.23468	Sterebin E	Naphthofurans	1.07	432.11
pos	347.22500	(13*R*,14*R*)-8-Labdene-13,14,15-triol	Diterpenoids	1.00	55.84
pos	345.24039	Darutigenol	Diterpenoids	1.32	1,319.91
neg	319.22714	11-HETE	Hydroxyeicosatetraenoic acids	1.2	1,748.88
neg	317.21173	9-HEPE	Hydroxyeicosapentaenoic acids	1.26	1,176.35
neg	317.2117	12-OxoETE	Long-chain fatty acids	1.31	3,736.83
pos	303.23229	Abietic acid	Diterpenoids	1.05	344.39
pos	291.26828	8alpha-13 [16],14-Labdadien-8-ol	Diterpenoids	1.25	6,568.23
pos	287.23642	Copalic acid	Diterpenoids	1.12	623.00
neg	215.16550	12-hydroxydodecanoic acid	Medium-chain hydroxy acids and derivatives	1.47	2,593.90
neg	199.17056	Dodecanoic acid	Medium-chain fatty acids	1.60	4,731.08
neg	185.15446	Undecanoic acid	Medium-chain fatty acids	1.32	363.84
neg	171.13928	Decanoic acid	Medium-chain fatty acids	1.46	5,471.94
pos/neg	167.10756	Chrysanthemic Acid	Monocyclic monoterpenoids	1.53	4,858.84
pos	151.11201	Thymol	Aromatic monoterpenoids	1.05	593.15
pos	135.11659	Carveol	Menthane monoterpenoids	0.92	255.34

pos, positive mode and neg, negative mode.

### The effects of coriander leaf essential oil on macrophage function and inflammatory signaling pathways during *C. albicans* infection

**Fig 2:**
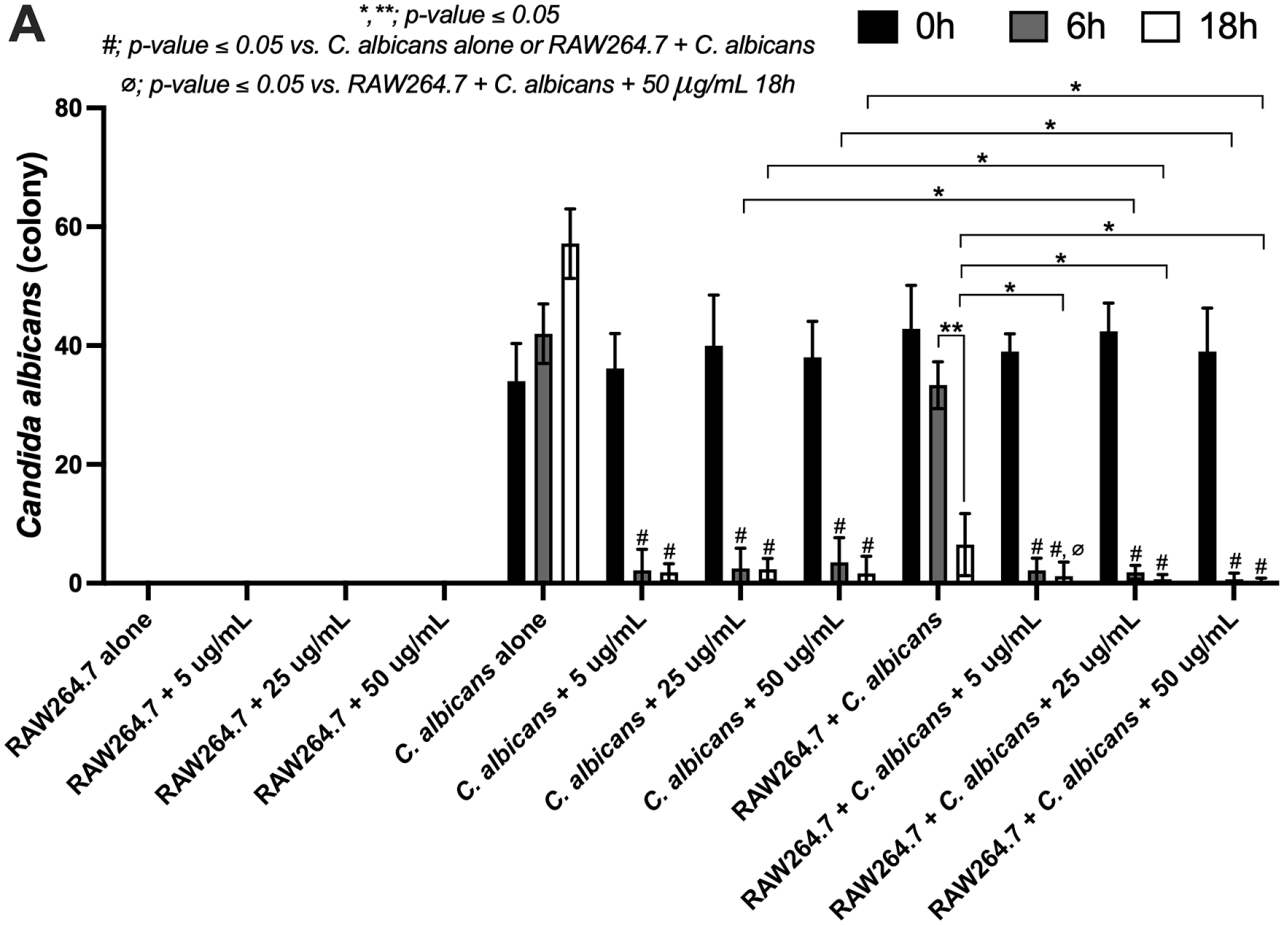
The characteristics of the RAW264.7 macrophage cell line after activation by *C. albicans*, either in the absence or presence of coriander essential oil (0 − 50 μg/mL), are demonstrated based on killing activity analysis. Data were collected in triplicate. The data are shown as the mean ± SE, *, **; *p-value ≤ 0.05*, #; *p-value ≤ 0.05* vs. *C. albicans* alone or RAW264.7 + *C. albicans*, and ⌀; *p-value ≤ 0.05* vs. RAW264.7 + *C. albicans* + 50 μg/mL 18 h, between the indicated groups using ANOVA with Tukey’s analysis.

Due to their involvement in host defense mechanisms against fungal pathogens, the ability of macrophages to exert killing activity and cytokine production was evaluated. The results demonstrated that coriander leaf essential oil at concentrations in the range 5–50 µg/mL significantly reduced the number of *C. albicans* colonies at both 6 and 18 h compared with the untreated *C. albicans* control group (*p ≤ 0.05*). Furthermore, when coriander leaf essential oil was administered in conjunction with RAW264.7 cells, a statistically significant reduction in *C. albicans* colony count was observed compared to the untreated co-culture group (RAW264.7 + *C. albicans*). This reduction displayed a dose-dependent trend. Notably, coriander leaf essential oil at a concentration of 50 µg/mL significantly enhanced the killing activity of RAW264.7 cells against *C. albicans* (*p ≤ 0.05*) (Fig 2A).

**Fig 3:**
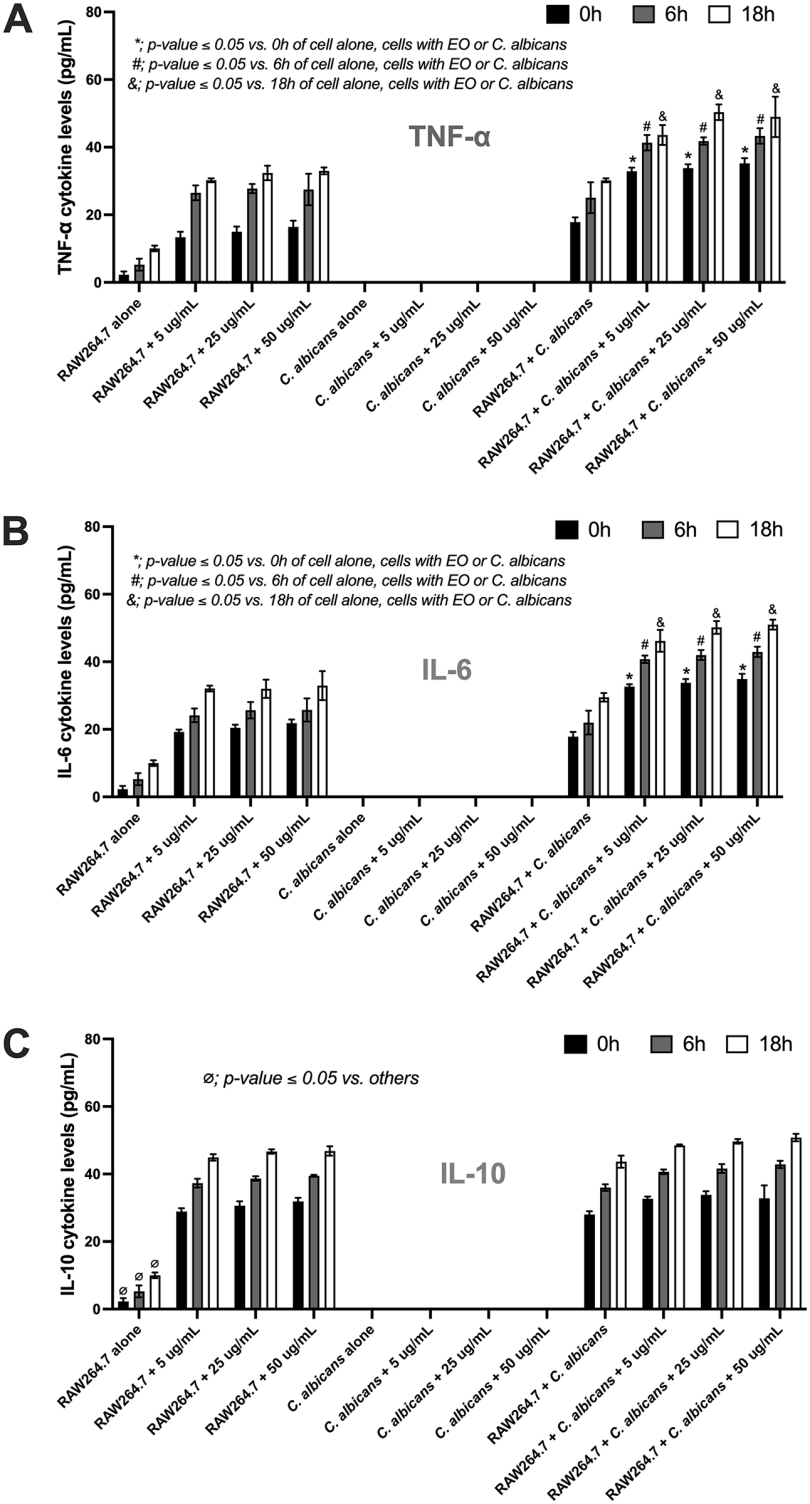
The characteristics of the RAW264.7 macrophage cell line after activation by *C. albicans*, either in the absence or presence of coriander essential oil (0 − 50 μg/mL), are demonstrated based on cytokine levels of TNF-α (A), IL-6 (B), and IL-10 (C) in the supernatant. Data were collected in triplicate. The data are shown as the mean ± SE, *; *p-value ≤ 0.05* vs. 0 h of other conditions, #; *p-value ≤ 0.05* vs. 6 h of other conditions, &; *p-value ≤ 0.05* vs. 18 h of other conditions, and ⌀; *p-value ≤ 0.05* vs. others, between the indicated groups using ANOVA with Tukey’s analysis.

The production of pro-inflammatory cytokines indicates that coriander leaf essential oil induces an immune response with an additive effect, as evidenced by elevated levels of both TNF-α (Fig 3A) and IL-6 (Fig 3B) compared with the other conditions. Briefly, administration of coriander leaf essential oil (5–50 μg/mL) to RAW264.7 cells during *C. albicans* infection resulted in a significant increase in TNF-α production. Specifically, treatment with 5 μg/mL coriander leaf essential oil significantly increased TNF-α levels at 0 h (32.90 ± 1.07 pg/mL; *p < 0.0001*), 6 h (41.33 ± 2.29 pg/mL; *p = 0.0009*), and 18 h (43.57 ± 2.95 pg/mL; *p = 0.0005*). Similarly, treatment with 25 μg/mL coriander leaf essential oil led to significantly elevated TNF-α production at 0 h (33.83 ± 1.07 pg/mL; *p < 0.0001*), 6 h (41.80 ± 1.09 pg/mL; *p = 0.0013*), and 18 h (50.32 ± 2.30 pg/mL; *p < 0.0001*). Treatment with 50 μg/mL coriander leaf essential oil also resulted in significant increases in TNF-α levels at 0 h (35.23 ± 1.51 pg/mL; *p < 0.0001*), 6 h (43.33 ± 2.29 pg/mL; *p = 0.0005*), and 18 h (48.91 ± 5.98 pg/mL; *p = 0.0022*). These TNF-α levels were significantly higher than those observed in the untreated co-culture group, which showed TNF-α concentrations of 17.83 ± 1.39 pg/mL at 0 h, 25.07 ± 4.57 pg/mL at 6 h, and 30.15 ± 0.59 pg/mL at 18 h.

The treatment also resulted in significant elevations in IL-6 levels. In brief, the treatment with 5 μg/mL coriander leaf essential oil significantly increased IL-6 production at 0 h (32.67 ± 0.66 pg/mL; *p < 0.0001*), 6 h (40.75 ± 1.11 pg/mL; *p = 0.0003*), and 18 h (46.20 ± 3.24 pg/mL; *p < 0.0001*). Similarly, treatment with 25 μg/mL coriander leaf essential oil led to significantly elevated IL-6 levels at 0 h (33.78 ± 1.10 pg/mL; *p < 0.0001*), 6 h (42.00 ± 1.48 pg/mL; *p = 0.0001*), and 18 h (50.17 ± 1.88 pg/mL; *p < 0.0001*). Treatment with 50 μg/mL coriander leaf essential oil also resulted in significant increases in IL-6 production at 0 h (34.92 ± 1.52 pg/mL; *p < 0.0001*), 6 h (42.91 ± 1.52 pg/mL; *p = 0.0003*), and 18 h (51.00 ± 1.48 pg/mL; *p < 0.0001*). These IL-6 levels were significantly higher than those observed in the untreated co-culture group, which exhibited IL-6 concentrations of 17.83 ± 1.39 pg/mL at 0 h, 22.00 ± 3.52 pg/mL at 6 h, and 29.50 ± 1.26 pg/mL at 18 h. However, no significant differences were observed across the time points or coriander leaf essential oil concentrations between 5 and 50 μg/mL. Additionally, the levels of the anti-inflammatory cytokine IL-10 also showed no significant differences among the tested conditions (*p ≤ 0.05*) (Fig 3C).

**Fig 4:**
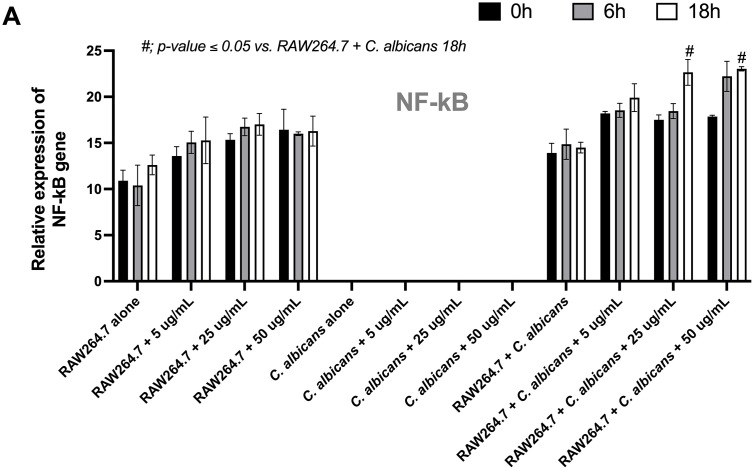
The characteristics of the RAW264.7 macrophage cell line after activation by *C. albicans*, either in the absence or presence of coriander essential oil (0 − 50 μg/mL), demonstrate the role of inflammatory responses based on *NF-κB* gene expression levels. The experiment was conducted in triplicate. The data are shown as the mean ± SE, #; *p-value* 0.05 vs. RAW264.7 + *C. albicans* 18 **h.**

Because immune cell activity is regulated through gene expression, NF-κB gene expression was analyzed. The results demonstrated an additive effect of coriander leaf essential oil, particularly at 18 h post-treatment. Treatment with 25 μg/mL coriander leaf essential oil resulted in a 1.5-fold increase in NF-κB expression (*p = 0.0106*), as same as treatment with 50 μg/mL also produced a 1.5-fold increase (*p = 0.0067*) compared with the untreated *C. albicans* control group (Fig 4A). However, no significant differences in NF-κB gene expression were observed among the different concentrations of coriander leaf essential oil (5–50 μg/mL) or between the different exposure times.

## Discussion

Over the past decade, essential oils have attracted considerable interest as a rich source of bioactive compounds with numerous potential health benefits. The essential leaf oil extracted from coriander leaf has been found to contain diverse bioactive constituents. The major monoterpenoids identified include thymol, carveol, and chrysanthemic acid, all of which have demonstrated antimicrobial and antioxidant activities [22,23]. Additionally, coriander leaf oil also contains several diterpenoid and triterpenoid compounds, including darutigenol, lagochilin, amyrin, and abietic acid, classified within the labdane and abietane structural groups commonly found in plants [24]. While their molecular structures have been characterized, common names and detailed bioactivities for many of these compounds are still lacking in current databases, so further studies are required. In terms of lipid composition, coriander leaf essential oil contains several saturated fatty acids, including lauric acid, capric acid, undecanoic acid, dodecanoic acid, and tridecanoic acid. In addition, hydroxydodecanoic acid, a hydroxylated saturated fatty acid, is also present. Notably, previous studies have reported that saturated fatty acids can modulate dendritic cells through Toll-like receptor 4 (TLR4) [25,26]. Furthermore, oxygenated derivatives of long-chain polyunsaturated fatty acids, such as 11-HETE, 9-HEPE, and 12-OxoETE, are associated with modulating inflammation and host immune response [27,28]. Our results demonstrated that coriander leaf essential oil supports macrophages in reducing *C. albicans* colonies, in line with previous studies [13,29*]*. The coriander oil constituents might bind to ergosterol in the *C. albicans* cell membrane, increasing membrane permeability and causing membrane damage and cell death. Moreover, coriander leaf essential oil has been reported to disrupt *Candida* biofilm and adherence [13]. These findings highlight the direct antifungal potential of coriander leaf essential oil, and further studies should be conducted to confirm these mechanisms.

Additionally, the coriander oil can modulate host immunity. For example, treatment of murine RAW264.7 macrophages with coriander seed oil significantly modulates immune responses through increased phagocytic and killing activity [29], suggesting an enhanced innate immune response. The direct microscopic visualization of macrophage engagement with, and killing of, *Candida* yeast cells would provide more robust evidence; therefore, future studies should incorporate such approaches. Aligning with these results, this study has shown that coriander leaf essential oil enhances macrophage immune responses, as evidenced by the enhanced production of pro-inflammatory cytokines (TNF-α and IL-6). These cytokines play crucial roles in promoting immune responses against fungal pathogens, including *C. albicans* [30,31]. Our findings suggest that an immune upregulation is associated with the activation of the NF-κB signaling pathway, a key regulator of inflammatory gene expression [32,33]. Mechanistically, the interaction between pathogen-associated molecular patterns (PAMPs), such as β-glucans in *Candida* cell walls, and pattern recognition receptors (PRRs) on macrophages initiates intracellular signaling cascades [34,35]. Among these receptors, Dectin-1 is a well-characterized C-type lectin receptor that specifically binds to β-1,3-glucans [36]. Upon ligand engagement, Dectin-1 initiates downstream signaling through spleen tyrosine kinase (Syk), leading to the activation of the CARD9-BCL10-MALT1 complex [37]. Leading to the phosphorylation and nuclear translocation of *NF-κB* subunits, facilitating the transcription of pro-inflammatory cytokine genes, including TNF-α and IL-6 [38,39]. Our data suggest that coriander leaf essential oil enhances this process, either by increasing the sensitivity of macrophages to β-glucan stimulation or by directly modulating intracellular signaling pathways. However, in the present study, only a single primer pair was used to validate the data. To achieve a higher level of confidence and to provide additional mechanistic insight into *Candida*-induced immune responses, additional primer sets and other inflammation-related genes should be investigated in future studies. In addition, bioactive constituents of coriander leaf essential oil, such as thymol and carveol, have been reported to exert immunomodulatory effects, potentially through NF-κB transcription factors [40]. It is feasible that these active compounds act synergistically to promote macrophage activation during encounters with fungi. Future studies should investigate the functions of these specific active compounds in modulating immune cell responses during *C. albicans* infection.

Interestingly, despite the enhanced pro- and anti-inflammatory cytokine productions and gene expression, no significant differences were observed across varying concentrations of coriander leaf essential oil (5–50 μg/mL) or between different exposure times. These results may indicate a saturation threshold in Dectin-1/NF-κB axis activation [39], suggesting that even low doses of coriander essential oil are sufficient to trigger maximal response. Furthermore, the observed immune stimulation might have additive effects when combined with fungal β-glucans [18,41]. Coriander leaf essential oil could function as a priming agent, sensitizing macrophages to fungal PAMPs, thereby amplifying Dectin-1-mediated *NF-κB* activation. Collectively, these findings support the potential of coriander leaf essential oil as a natural immunomodulator that enhances macrophage responses via the NF-κB pathway. This mechanism may be relevant not only for fungal clearance but also for broader applications in immunotherapy and inflammation regulation, warranting further *in vivo* investigation to clarify its mechanisms and therapeutic potential.

## Conclusion

This study highlights the potential of coriander leaf essential oil as a natural immunomodulatory agent with antifungal activity against *C. albicans*. The findings demonstrate that coriander leaf oil had an additive effect that can increase macrophage responses, particularly through modulation of killing activity, cytokine production, and the *NF-κB* gene expression. These results suggest that coriander essential oil may serve as a promising adjuvant in antifungal therapy, supporting host immune defense while exerting direct antifungal effects.

## Supporting information

S1 File Cytokines. (XLSX)

S2 File LC-MS_Coriander Leaf Oil. (XLSX)

S3 File NF-kB gene. (XLSX)
